# Feasibility of a randomized controlled trial of a proprioceptive and tactile vest intervention for children with challenges integrating and processing sensory information

**DOI:** 10.1186/s12887-022-03380-5

**Published:** 2022-06-02

**Authors:** Ann Natasja Nielsen, Karen la Cour, Åse Brandt

**Affiliations:** 1 National Institute of Public Health, Research Unit for Child and Adolescent Health, University of Southern Denmark, Copenhagen, Denmark; 2 Department of Public Health, Research Unit for User Perspectives and Community-based Interventions, University of Southern Denmark, Odense, Denmark

**Keywords:** Sensory processing, Sensory integration, School, Proprioceptive-tactile stimulation

## Abstract

**Background:**

Children with challenges integrating and processing sensory information can have difficulties participating in play and learning activities. One way to support participation is to offer sensory stimulation, such as proprioceptive and tactile stimulation provided by wearing a sensory-stimulating vest. The aim of this study was to investigate the feasibility of the key procedures of a planned full-scale randomized controlled trial (RCT) of a proprioceptive and tactile stimulation vest for children aged 6-12 years who have challenges integrating and processing sensory information. The study focused on the feasibility of the recruitment and information processes, the relevance of the study materials, the usefulness of diaries completed by parents, and use of the following outcome measures: Test of Everyday Attention-Children (TEACh), registration of off-task behaviour, and pulse rate.

**Methods:**

Ten schoolchildren aged 6–12 years (mean 8.4) who had challenges integrating and processing sensory information and sensory-seeking behaviours (based on their Short Sensory Profile scores) participated in the study. Study feasibility was assessed using data from interviews with the participating children, their parents and teachers, and psychologists from the municipal Educational Psychological Counselling Departments.

**Results:**

Recruitment and introductory materials were found to be relevant and non-problematic, while the outcome measurements, diaries, and pulse measurements did not work well, and the tool for registering off-task behaviours needed to be revised. The results indicated that an outcome measure relating to the children’s subjective experiences and closer involvement of teachers in the study could be beneficial.

**Conclusion:**

The aim of the study was to investigate the feasibility of the planned methodology for a full-scale RCT of a proprioceptive and tactile stimulating vests for children with challenges integrating and processing sensory information. We found that a partial redesign of the study is needed before a full-scale RCT is conducted and that this should include outcome measures on the children’s subjective experiences with using the vest.

**Supplementary Information:**

The online version contains supplementary material available at 10.1186/s12887-022-03380-5.

## Background

Children’s development is stimulated through dominating activities such as play and schoolwork, and a child’s ability to participate in these activities is thus an important basis for their development [1]. Sensory processing refers to the ability to regulate and organize sensory information in an adaptive and graded manner [2]. Children with challenges integrating and processing sensory information can have difficulties sitting still, paying attention, and holding focus [2, 3], and this can affect a child’s ability to concentrate [3] and learn while attending school [4]. The most common category of challenges integrating and processing sensory information is sensory-seeking behaviour such as frequent movement, fidgeting etc., which can interfere with a child’s ability to participate in activities [2]. Sensory challenges also affect children’s choice of and participation in activities [4–6], and children with challenges integrating and processing sensory information have significantly lower levels of participation and enjoyment in everyday activities, both in school and at home [7]. Additionally, childhood sensory processing difficulties may be linked to youth anxiety and adult obsessive-compulsive symptoms [6, 7].

Approximately 5-16% of American preschool children are estimated to have challenges integrating and processing sensory information [8], while this was the case for 19.9% of 141 Puerto Rican preschool children [9] and 21.3% of 1721 Danish primary school children [10].

**Fig. 1:**
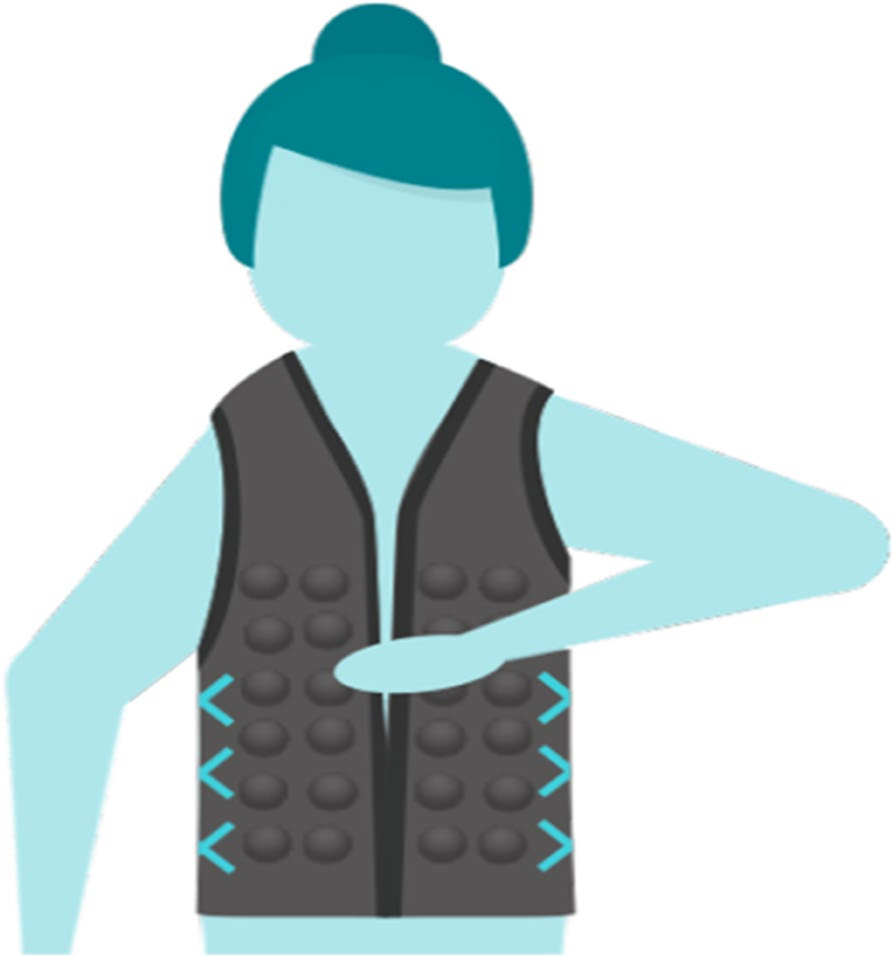
The Protac Myfit vest

A review of occupational therapy studies that used Ayres Sensory Integration® intervention for children with challenges integrating and processing sensory information found that improved sensory integration and processing had a positive effect on children’s concentration and social and reading skills as well as on their participation in play [11]. However, individual or group-based occupational therapy or physiotherapy intervention, consisting of Ayres Sensory Integration® [11] or sensory skills training [12], can be both time-consuming and expensive. Another way to support children with challenges integrating and processing sensory information is through the use of sensory-stimulating vests as a supplement or replacement. In the present study, the Protac Myfit® vest (here from referenced to as the vests) was used (Fig. 1). This is commercially available and is widely used in the Danish healthcare system. The vest has compartments running down the front and back that are filled with weighted plastic balls that provide proprioceptive and tactile stimulation. The vest is constructed from an elastic material and has a zipper at the front and adjustable drawstrings on the sides to allow the fit to be adjusted. The balls in the vest are either 25 mm or 38 mm in diameter, and the vest weighs 1–2.5 kg depending on its size.

A Swedish pilot study of children with attention deficit hyperactivity disorder who used the vest showed a positive effect on the children’s ability to concentrate and maintain attention after a 14-day test period. The study recommended further research into the effects of using the vests as well as clear recommendations for practice [13].

In Denmark, a large randomized controlled trial (RCT) is planned to compare the use of the vest with usual treatments/interventions provided by Danish municipalities for children with challenges integrating and processing sensory information. To ensure appropriate evaluation of the effects of the intervention and generation of relevant recommendations, the study will follow the guidance of the Medical Research Council (MRC) regarding complex interventions [14]. The MRC considers complex interventions to be studies with a range of intervention elements and outcome variables [14]. When developing and evaluating complex interventions, an important element is to thoroughly test the feasibility of an evaluation study [14].

### The planned RCT

The RCT would be carried out in collaboration with multiple municipalities across Denmark; more specifically, with the Educational Psychological Counselling Departments (EPCD) of the municipalities. EPCD is an interdisciplinary support function that advises institutions and schools on children aged 0–18 years.

The participating children and parent would be recruited and informed about the study through materials distributed by the EPCD. These materials would include an introductory pamphlet, a study-specific questionnaire on demographics (the child’s age, gender, school, school grade, diagnoses, and municipality) and the Danish version of the Short Sensory Profile (SSP) questionnaire, which would be used for identifying challenges integrating and processing sensory information and sensory-seeking behaviour. The SSP is a validated 38-item screening tool in which parents score statements about their child’s behaviour on a five-point Likert scale in relation to how often they observe their child performing the described behaviour [15]. The SSP gives a total score between 38 and 190; a score of 155–190 indicates typical presentation in integrating and processing sensory information, and a score of 38–141 (scores at least 2 SD from the mean) indicates that the child has challenges integrating and processing sensory information [15]. The SSP items are grouped into seven different behavioural sections: *Tactile Sensitivity* (7 items), *Taste/Smell Sensitivity* (4 items), *Movement Sensitivity* (3 items), *Under-responsive/Seeks Sensations* (7 items), *Auditory Filtering* (6 items), *Low Energy/Weak* (6 items), and *Visual/Auditory Sensitivity* (5 items). A score of 7–23 in the section on *Under-responsive/Seeks Sensations* indicates that the child has sensory-seeking behaviour.

- Criteria for inclusion: Primary school children aged 6-12 years who are referred to EPCD due to a need for support to maintain attention while in school and have challenges integrating and processing sensory information and sensory-seeking behaviour, established by means of the Short Sensory Profile (SSP).
- Criteria for exclusion: Children with intellectual developmental disabilities or cognitive impairment, and children using a corset or a moulded wheelchair.

The eligible participants would be randomized (1:1) via a computer-generated algorithm into an intervention and a control group. The parents would be informed by phone whether their child had been randomized to the intervention or control group. At the end of the study period, the children in the control group would be offered the opportunity to use the vests over a three-week period.

Both the intervention and the control group would receive usual treatment, but the intervention group would also use the vest. At baseline, the vest would be adjusted using the drawstrings to fit the child. Additionally, the child would decide which ball size (25 mm or 38 mm in diameter) was most comfortable in the vest. The child and the parents would be thoroughly introduced to the use of the vest (i.e. the parents by phone at enrolment, and the children in person at baseline). The children would be encouraged to use the vest as much as they liked over a three-week period both at home and in school. Parents would be asked to have conversations with the children about the vest and then to document the child’s use of and experience with the vest using a diary containing prewritten questions about the use of the vest and other treatment; parents would also be encouraged to use the diary to write down their own reflections about the use of the vest (e.g. positive and negative effects or obstacles to its use).

Teachers of the children in the intervention group would receive an introductory pamphlet about the study, and the teachers would be encouraged to explain the study to the children’s classmates with the intention of reducing the possibility of stigmatization.

The primary outcome would be the child’s level of attention as measured by the Test of Everyday Attention-Children (TEA-Ch). TEA-Ch is a standardized battery of nine tests that examine three different types of attention (selective attention, sustained attention, and attentional control/switching). The TEA-Ch is designed for retesting with two parallel versions, version A for baseline and version B for follow-up. The TEA-Ch subtest results are assessed independently, and the age- and sex-specific scale scores range from 1 to 19 [16].

Secondary outcomes would be the child’s frequency of off-task behaviour and any calming effect. Off-task behaviour in school would be measured using a study-specific observation tool comprising a registration form and manual. The registration form would include 10 items, each describing a specific off-task behaviour. The researcher would observe the child for 30 minutes during a teaching session at school and mark every time the child performed the behaviours described in the 10 items. Calming effect would be measured by pulse rate as mental and physical stress typically causes the pulse rate to rise [17]. To examine the child’s pulse rate, a pulse oximeter and smartwatch would be used during the first 30 min of the TEA-Ch testing.

## Methods

The aim of the current study was to investigate the feasibility of the planned full-scale RCT described above, i.e., to investigate the feasibility of the key procedures of an RCT of a proprioceptive and tactile stimulation vest for children aged 6-12 years who have challenges integrating and processing sensory information as well as sensory-seeking behaviour.

### Specific objectives

A. To investigate the recruitment process and the distribution of recruitment material.
B. To investigate the relevance to parents of the text in the introductory materials, recruitment material, and questionnaire.
C. To investigate the information process with parents regarding participation in the study and experiences of being included in either the control or intervention group.
D. To investigate the usability of parental diaries.
E. To investigate experiences of the children using the vest.
F. To investigate the feasibility and practicality of the test situation. Specifically, the children’s ability to switch between different settings (observation in class, testing and fitting of the vest)
G. To investigate the relevance and usability of the selected outcome measures.

For an overview of the specific objectives and data collection, see Table 1.

**Table 1:** Overview of the specific objectives and data collection of the current study

Specific objectives	Method for data collection	Source
A. Recruitment	Semi-structured interviews Internal and external dropout analysis	EPCD psychologists
B. Materials to parents	Semi-structured phone interviews	Parents
C. Information about inclusion and exclusion	Semi-structured phone interviews	Parents
D. Usability of diaries	Semi-structured phone interviews Comments in the diaries	Parents
E. Experiences of the children using the vest	Semi-structured phone interviews Diaries Semi-structured interviews E-mail	Parents Children EPCD psychologists
F. Test situation	Semi-structured interviews Semi-structured interviews	Children Teachers
G. Outcome measure	Semi-structured interviews Observations	Children Researcher

### Study design

The study was designed as a feasibility trial that aimed to investigate the feasibility of key procedures for a full-scale RCT. The study was conducted in the same way as the planned full-scale RCT with some additional data collection for the feasibility trial as described below. The study was carried out at three schools in a Danish municipality in collaboration with their EPCD.

### Participants

Ten children fulfilling the inclusion criteria for the planned RCT were included in this feasibility study. Other study participants were psychologists working in the EPCD and the children’s parents and teachers.

### Methods

The inclusion and exclusion criteria for the feasibility study were the same as those for the full-scale RCT, as were the intervention, outcomes measured, and tools used. Specific methods for the feasibility study are presented below according to the different stages of the study.

### Recruitment

The municipality that participated in the feasibility study only had psychologists working in the EPCD. Therefore, psychologists from the EPCD affiliated at different schools throughout the municipality recruited children to participate in the study. The director of the EPCD sent instructions to the psychologists regarding the department’s participation, a written introduction to the study, and information about challenges children can have with integrating and processing sensory information and sensory-seeking behaviour. The psychologists were given several time slots where they could talk to the researcher about challenges related to integrating and processing sensory information and could discuss any questions or uncertainties about the study. The psychologists were subsequently given recruitment materials to give to parents of children who they thought might be eligible for the study. Parents filled out the demographic- and SSP questionnaires and consent form and sent it to the researcher per mail.

### Randomization

The children who met the inclusion criteria were randomized using sequentially numbered envelopes (1:1) into two groups: Group A (*n* = 5) was the intervention group and Group B (*n* = 5) was the control group.

### Intervention and control

The intervention group received the vest and explanations as described above for the planned full-scale RCT. The control group received typical EPCD treatment, which consisted of supervision or advice given to the teachers from the psychologists.

### Data collection

Data were gathered through qualitative interviews and diaries. An overview of data collection in relation to the specific objectives is presented in Table 1.

### Interviews

All interviews were conducted in Danish by the first author and were based on semi-structured interview guides developed for the study and directed to the specific participant group (i.e. psychologists, children, parents, and teachers). The interviews lasted between 5 and 15 minutes. An English version of the interview guide is included as a supplementary file (Additional file 1). All interviews were conducted in person except for those with parents, which were conducted over the phone.

Interviews with the psychologists were based on an interview guide with themes concerning the information given to them about the study and their expectations regarding participation. The interviews were conducted after the psychologists had been introduced to the project.

Interviews with the children were based on an interview guide that explored how the children experienced using the vest, the test situation, and the usability of the pulse-measuring devices. These interviews were carried out at baseline and at follow-up.

Interviews with the teachers were based on an interview guide with open-ended questions about their experience with the children’s use of the vest and whether there was consistency between their expectations based on the materials given and their experiences. These interviews were performed after the follow-up with the children.

Parents were interviewed twice by phone. The first interview was performed in connection with the call informing them about their child’s inclusion or exclusion in the study. This interview focused on the information material, the demographic questionnaire, the SSP, communication about the inclusion/exclusion process, and randomization into group A or B. The second phone interview was performed the day before follow-up. The interview guide focused on the parents’ experiences of their child’s use of the vest, the usability of the diaries, and whether there was consistency between the parents’ expectations about participating in the study and their experiences from actually participating in the study.

### Diaries

The diaries had daily prewritten questions about whether the child had used the vest in school and/or at home (as judged by parents from their conversations with their child), and whether the child had received any additional treatment. The diary also had a comment section where parents were asked to write down their reflections of and experiences with the child’s participation in the study and use of the vest.

### Additional forms of data collection

At the end of the study, the psychologists were contacted via e-mail, asking them to reflect on their experiences during the study and to provide any feedback on information regarding challenges integrating and processing sensory information and the process of recruitment. They were specifically asked about any motivating or demotivating factors they experienced when recruiting participants to the study.

For internal and external dropout analysis, the recruitment materials distributed were counted (i.e. how many sets of materials were handed out to parents, and how many sets were returned from the parents).

### Data analysis

Qualitative data from the interviews were subjected to thematic analysis by the main author according to the predefined objectives regarding procedure and feasibility [18]. Quantitative data received from parental diaries were summarized to examine the amount of use of the vest. Internal and external dropout analysis was performed by counting both the number of materials distributed and the number of materials returned. Descriptive statistics (mean and standard deviations) were used to present the test results from TEA-Ch.

## Results

The participants in the feasibility study were 10 children (eight boys and two girls) aged 6-12 years (mean 8.4 years, SD 1.3) who attended three different public schools (three in grade zero, six in grade two, one in grade four). The SSP total scores of children tested for eligibility are presented in Table 2.

**Table 2:** Presentation children tested for eligibility

Sex	Total Short Sensory Profile score	Section score for “Under-responsive/Seeks Sensations”^a^
Included		
Boy	118	14
Boy	116	14
Boy	129	17
Boy	148	15
Boy	138	20
Girl	142	20
Girl	145	23
Boy	133	19
Boy	134	19
Boy	127	21
Excluded		
Girl	173	29
Boy	158	25

^a^According to the Short Sensory Profile

The results are presented according to the specific objectives.

### The recruitment process and distribution of recruitment material

Five out of six psychologists chose to participate in the study and the question time, where they asked questions about; challenges integrating and processing sensory information, the recruitment materials, and discussed examples of children’s behaviour they had experienced and were wondering if might be due to challenges integrating and processing sensory information. The sixth psychologist declined to participate in the study as she was new to the EPCD and felt that she did not have the necessary insight into the children at the school to participate in the study.

Feedback given by the participating psychologists during the interviews suggested that they were positive about the study and the introduction material.

All showed interest in the subject of challenges related to integrating and processing sensory information among children, and four expressed a wish for additional support to the schools, such as occupational therapy or aids such as the vest. For example, one psychologist expressed this need about a child that she suspected might have challenges integrating and processing sensory information: “I don’t know what to do with him, perhaps you people (occupational therapists) can help”. Another psychologist expressed: “Great that we can talk it over, we need you people out at the schools”. One psychologist expressed concern about parents’ ability to understand and complete the questionnaire without help, especially those with language barriers or dyslexia. Additionally, she expected that some parents might get frustrated with her if their child was not included in the study.

The analysis suggests that it was beneficial to have the question time where the psychologists could ask questions and discuss potential study participants. None of the participating psychologists chose to answer the evaluation e-mail or give additional feedback at the end of the study, indicating that these approaches are not feasible.

Twenty-one sets of parental questionnaires (i.e. SSP and demographics) were handed out at three of the five schools; of these 12 sets questionnaires were returned, indicating that the information in the introduction and recruitment materials was sufficient. The management at these three schools contacted the researcher and expressed interest in the study and the subject of children with challenges integrating and processing sensory information. A further 35 sets of parental questionnaires were given to the two remaining psychologists, but it is not known how many of these were handed out to parents due to lack of responses at the end of the study. This seems to indicate that involvement of school management could be beneficial for a full-scale RCT.

### The relevance to parents of the introductory and recruitment material and the questionnaire

Out of the 12 parental questionnaires received, 10 showed a total SSP score < 141 (indicating challenges integrating and processing sensory information) and a section score < 23 (indicating sensory-seeking behaviour). This indicates that the introduction material was relevant and adequate to identify appropriate participants for the study. All of the 10 children who scored < 141 were included in the study. The total SSP scores for the other two children (who were not included in the study) showed typical presentation.

When directly asked about their perception of the material, most parents were positive. One expressed: “It was fine, but there were a lot of slightly strange questions, but we answered as well as we could”. When asked which questions she found strange, she mentioned items related to the SSP. Another said: “It looked a bit like advertising (referring to the introductory material) ….. Does he need to wear the vest every day”?

Some parents did not seem especially interested in the study and gave the impression that they had only participated because of the teacher’s recommendation, indicating that a closer involvement of the teachers could be beneficial. One parent expressed it this way: “The teacher thinks it is a good idea, so yes, he can try it”.

Both the introductory material and the recruitment letter indicated that study participation would only comprise a three-week try-out of the vest. During the second interview, however, one parent expressed frustration that the study did not meet her expectation that her child would be provided with the vest after the study ended, indicating that this should be expressed more clearly.

### The information process to parents regarding exclusion and inclusion in control or intervention group

Parents of included children were friendly and forthcoming during the phone calls about their children’s participation in the study. Parents of children in the control group had no complaints about having to wait 3 weeks before trying the vest.

The parents of the two children excluded from the study had different reactions. One was happy to find out that her child did not appear to have challenges integrating and processing sensory information according to the SSP. The other child’s parent was very sad and asked if there was anything, she could do to get her child included in the project even though the child did not meet the inclusion criteria. She mentioned that her child had been in a previous study where the child got to use a ball blanket and it helped with sleep difficulties, but due to grant issues they did not get to keep the blanket.

### The usability of parental diaries

The entries in the diary ranged from 1-11 days, and none of the parents wrote in the diary daily over the entire three-week period. During the second phone interview, when the parents were asked about why they had not used the diary throughout the entire three-week period, the most common explanation was that they forgot. But during the follow-up phone interview, it emerged that some of the parents believed that the project was a school issue; one parent explained that her son had been happy to wear the vest in school, but: “he almost never brought it home with him”. One set of parents had given the diary to the teacher to fill out; this diary was only filled out 8 days and, according to the primary teacher, it was because: “the other [teachers] don’t take responsibility”, i.e. the rest of the child’s teachers had not wished to complete it. This demonstrates that a closer involvement of teachers could be beneficial for the study.

The diary had a daily open question about reflections regarding the study; this question helped to identify that two of the participants did not enjoy wearing the vest. One diary entry reported that it was because the vest irritated the child’s back when sitting in his chair in class: “He doesn’t like how it feels on his neck”. No additional treatment for any of the included children was documented within the study period.

### The experiences of the children using the vest

Due to limited diary entries, it is unknown exactly how much the children used the vest. Interviews with the parents suggested that a more structured schedule for the use of the vest could be beneficial; one parent expressed that the child had an agreement with the school to use the vest at the beginning of every school day, and another parent that: “She used it every day in school, according to her agreement with the teacher”. Interviews with the children also showed that support from teachers influenced their use of the vest; one child expressed “sometimes [teachers name] tells me to find it”. Another boy did not like the vest and called it: “stupid and ugly”. When asked why he had worn it every day, he answered that it was because it made him better at the computer game he played as part of the school lesson. This shows that a close involvement of teachers to support children could be beneficial. Results from interviews with the children showed that the children assessed their experiences of using the vest critically, some described experiences of internal feelings of calmness, some had experienced that using the vest affected their behaviour and some that the vest did nothing for them. One boy stated, “it was fine and cool, but I could not really feel a difference”. The children described their experiences with the vest also with considerations for their surroundings, one girl stated, “when I am wearing the vest, I can sit at the big table with the other children without disturbing them”.

### The feasibility and practicality of the test situation

The children were prepared for participating in the study. When one child was informed that we were going to do testing, he said: “Mom already told me”, and often the child had also reminded his or her teacher that the researcher was coming.

The children were all able to switch between the different settings (i.e. observations conducted in their classroom and testing in a meeting room at the school). All the children were able to contribute with specific feedback on different tasks during the interviews, such as whether they preferred the oximeter or the smartwatch for measuring pulse: “It’s a bit annoying that we have to stop (to put on the oximeter and note the pulse)” or how it was to try out the vest: “It’s very cool” or “It’s nice…especially the one with big balls… I almost can’t feel the small ones”.

### The relevance and usability of the selected outcome measurements

#### TEA-Ch

Six out of the 10 children were able to complete all nine of the TEA-Ch subtests at both baseline and follow-up; a further two children were able to do eight subtests at baseline, and one of these did all nine subtests at follow-up. It takes approximately 1 h to complete all nine subtests. Among the four children who were not able to complete all nine subtests, two children found it too challenging to sit still long enough to complete the test. One child could complete five of the subtests but found it very difficult: “It’s just very difficult… I have never tried it before”, and one child only completed the first subtest, then said: “I don’t want to do it anymore… the numbers are getting away from me”. These two children both had emotional reactions to the tests and the challenges they presented. Both children were attending a class at the school with a specialized and protected environment. Hence, children from protected classes seem to find the test too difficult and should thus not be included in the full-scale RCT.

The children that completed all subtests were engaged in the tests and showed their engagement with smiles and interest in their scores. During the interview they were asked about their opinion of the tests, and the most common answer was: “It was fine”. One child described it: “It’s both a bit boring and a bit difficult”.

Results from the eight children who completed eight or nine of the subtests are presented in Table 3. The intervention group showed improvement in attention in six out of nine subtests whereas the control group showed improvement in four subtests.

**Table 3:** Means with standard deviations of Test of Everyday Attention-Children (TEA-Ch) scores (Intervention group *n* = 4, control group *n* = 4). Higher scores reflect better attentive abilities

Domain	Subtest	Intervention group baseline	Intervention group follow-up	Control group baseline	Control group follow-up
Sustained attention	Score	5.5 (1.7)	7 (1.9)	10 (4.8)	8 (4.4)
	Sky Search DT	4.5 (8.5)	8 (3.7)	4.5 (5.1)	6 (6.1)
	Score DT	6.5 (1.7)	7 (4)	7 (5.6)	6 (5)
	Walk, Don’t Walk	9 (2.9)	7.5 (2)	5 (2.6)	6 (3.2)
	Code Transmission	5.5 (3.3)^a^	6 (1.5)	6.5 (5.7)^a^	4.5 (6.1)^a^
Selective attention	Sky Search	8 (3.7)	7.5 (2.5)	5.5 (3.6)	9 (3)
	Map Mission	6 (1.8)	7.5 (1.9)	8 (3)	7.5 (3.6)
Attentional control	Creature Counting	10 (3.8)	9 (3)	7 (2.6)	5 (4.3)
	Opposite Worlds	6.25 (1.9)	8 (1.7)	8 (3.9)	8 (3.5)

^a^*N* = 3

#### Pulse

Using the oximeter for measuring the pulse was distracting because the children had to put in on and take it off every time the pulse was to be measured. One child expressed: “It’s annoying that we have to stop so much, start and stop all the time”. Therefore, the oximeter was discarded after eight baseline tests and two follow-up tests and only the smartwatch was used hereafter. The children’s pulse rates increased dramatically due to the excitement of the different tests that they were doing, resulting in pulse fluctuations between 62 and 120 beats per minute. Thus, pulse was found not to be a relevant outcome measurement.

#### Registration of off-task behaviour

It was not possible to complete the registration of off-task behaviours as the registration could not be completed during two comparable observation sessions at the schools. This was due to setting changes such as sport events, substitute teachers, and a special project week. Although observations were carried out at agreed times and baseline and follow-up observations were performed at the same time on the same weekday 3 weeks apart, it was not possible to make comparisons between the two observation sessions.

#### Additional results

During the interviews with the children, it became apparent that some of them were experiencing low self-efficacy and self-esteem. For example, when asked, “Do you know why you are here?”, one boy said, “Because I’m a bad boy, that is bad at attending school”. According to his teacher and parents, this was not the case, but the boy was becoming more and more insecure and felt out of place and uneasy. After wearing the vest for 3 weeks, both at home and in school, one boy stated, “It’s good, very good…. It’s just nice to wear it”.

## Discussion

The primary aim of the current study was to investigate the feasibility of key procedures planned for a full-scale RCT of proprioceptive-tactile stimulation using the vest for children aged 6-12 years who have challenges integrating and processing sensory information as well as sensory-seeking behaviour. Results indicate that some aspects of this study were feasible to include in the full-scale RCT whereas others were not.

### Non-problematic procedures

Non-problematic aspects appeared to be the applicability and relevance of the introductory materials, recruitment letter, and questionnaire to the parents; the process of informing parents about whether their child was included or excluded from the study and, if included, whether their child was in the control group or intervention group; and the practical aspects of completing different tests and tasks at baseline. These materials and procedures can thus be used in the planned full-scale RCT.

### Procedures that were not productive

The use of parental diaries was not productive as most parents did not write in the diary. Research suggests that the use of diaries can be challenging, but they also help to gain a personal insight into the experiences of being part of the intervention [19]. Since the diary was not used very often, this could indicate that parents either did not find it relevant or did not have enough time to participate in the study at that level. Perhaps we had not chosen an appropriate format, or parents might be more inclined to answer the diary questions if this was done in closer collaboration with the teachers or they received a daily reminder.

Pulse as an outcome measure was found not feasible for this study due to how easily the child’s pulse was influenced by what they were participating in or what was going on around them. This is similar to findings in a study of children’s heart rates that found that pulse measured in community settings was higher than that measured in clinical settings [20]. Pulse is used as an outcome measure in other studies of products similar to the vest such as ball-blankets, but this has been in relation to sleep and measured through the night.

### Procedures needing amendments

Regarding the recruitment process, the distribution of recruitment materials and the introductory discussions with the psychologists were found to be time-consuming. Since all participants came from schools with engaged management, it became clear that the involvement of the school management could have an influence on participation. Additionally, it was clear that teacher involvement influenced the children’s participation and the use of the vest. Therefore, it is anticipated that a close partnership with management and teachers at the primary school level could benefit the study. According to the model for Pragmatic Explanatory Continuum Indicator Summary (PRECIS-2), a clinical trial should consider and map out where the design setup is on a pragmatic/explanatory continuum [21]. Adjusting the recruitment process to include more collaboration and a closer partnership with the individual schools would contribute to a more pragmatic setup as assistive devices such as the vest are usually provided by the schools or require close collaboration with school management.

Some of the outcome measurements should be modified. Not all children were able to complete the entire TEA-Ch, so the test could perhaps be shortened. Similar time challenges with using the full TEA-Ch were reported by Yochman et al. [22].

The use of observations to register off-task behaviour will need to be modified. Due to schools being an everchanging environment with different setting changes, such as special assignments and project/sports weeks, it was not possible to have two comparable observation sessions during the children’s usual classroom teachings. This was similar to challenges with school scheduling and time commitments found in a recent feasibility study by May-Benson and Teasdale [23]. An observational session could instead be held while the children complete the TEA-Ch. By performing the observation and TEA-Ch simultaneously, the test settings and results are expected to be more comparable.

Finally, it became apparent that the chosen outcome measures were not sufficient for exploring or identifying the children’s subjective experiences with having challenges integrating and processing sensory information or with using the vest. The children described their experiences in depth and with considerations for their own behaviour and their surroundings, but it was not possible to draw any conclusions from the feasibility. Therefore, such outcome measures should be added to a full-scale RCT. One boy described the vest as stupid and ugly, how this might have affected his use of the vest is unclear and should be further investigated. Additionally, a full-scale RCT should be focused on preventing possible stigmatisation of children wearing a vest that might be perceived as ugly or stupid. All adjustments should be feasibility tested in a second study before a full-scale RCT is conducted.

### Methodical considerations

As this was a feasibility study, the sample size was fairly small, and the results cannot be generalized. The SSP is a screening tool for research, where a full Sensory Profile could provide greater insight in the behavioural patterns of the children the SSP was found more relevant for the purpose of screening for eligibility. A limitation was the lack of responses to questionnaires sent to the psychologists; perhaps there would have been a higher response rate if a different method for data collection had been chosen. Another limitation was that all the written materials and interviews were in Danish, which was not the first language for all parents; this may have affected some parents’ engagement and participation in the study.

#### Ethics

All participants gave informed consent to participate in the study, and parents additionally gave written consent for the children to participate and use the vest. The study shed light on some ethical issues that a full-scale RCT should try to counter. One issue that became especially apparent was that the children in classes with protected environments had emotional reactions to the TEA-Ch tests. Therefore, it should be considered whether children from special classes should be included in the full-scale RCT. Additionally, the RCT should try to ensure that the children included in the study are able to handle participation in a study. This could be done in close collaboration with the children’s teachers.

It was noted that some parents had unmet expectations about whether participation in the study would result in the school providing vests for the children if they had benefitted from using the vests. This type of grant issue is similar to challenges experienced in another feasibility study of interventions for children with challenges integrating and processing sensory information [23]. The RCT will need to be more explicit in what can be expected regarding use of the vests at the schools.

## Conclusion

The aim of the current study was to investigate the feasibility of the planned methodology for a full-scale RCT of a proprioceptive and tactile vest for children with challenges integrating and processing sensory information. We found that a partial redesign of the study is needed to include outcome measures on the children’s subjective experiences with using the vest. Furthermore, an additional pilot or feasibility study is needed to investigate a modified recruitment strategy that includes school management and the addition of a reminder by e-mail or text to the parents instead of the diary. The tool for registering off-task behaviour will also need to be redesigned. However, the introductory materials, the information process with the parents, the questionnaire completed by the parents, and the practical aspects of completing the different tests were all acceptable and can be retained in the full-scale RCT.

## Supplementary Information

**Additional file 1.** Interview guides. English version of interview guides for the participating psychologists, children, parents, and teachers.

## Data Availability

Data are not available due to European laws on General Data Protection Regulation.

## Abbreviations

EPCD: Educational Psychological Counselling Departments
MRC: Medical Research Council
PRECIS: Pragmatic Explanatory Continuum Indicator Summary
RCT: Randomized controlled trial
SSP: Short Sensory Profile
TEA-Ch: Test of Everyday Attention-Children
