# Supplementary material for: Feasibility of a randomized controlled trial of a proprioceptive and tactile vest intervention for children with challenges integrating and processing sensory information
Source: BMC Pediatr. 2022 Jun 2;22:325. doi: 10.1186/s12887-022-03380-5 (PMC9161456; doi:10.1186/s12887-022-03380-5)
Supplement: Supplementary file 1 — Additional file 1. Interview guides. English version of interview guides for the participating psychologists, children, parents, and teachers. [file 12887_2022_3380_MOESM1_ESM.docx]

Interview guides

All interviews were conducted in Danish by the first author, based on these study-specific interview guides, directed to the specific participant-group (i.e., psychologists, children, parents, and teachers). All interviews were conducted in person except for the interviews with parents, which were conducted over the phone.

# Psychologists

Conducted subsequent to the psychologists’ introduction to the project.

| Objective | Theme question | Supplementary questions |
| --- | --- | --- |
| A: To investigate the recruitment process and distribution of recruitment material | Was the information given regarding sensory integration adequate? | - For you to select possible participants.  - For you to introduce parents and teachers to possible participants. |
|  | Do you find the project relevant for your school? | - Regarding the use of the vest  - More generally, the use of sensory tools. |
|  | What are your expectations to the project? |  |

Questions sent by mail at the end of the study

| Objective | Questions |
| --- | --- |
| A: To investigate the recruitment process and distribution of recruitment material | Did the study live up to your expectations? |
|  | Did the children you expected get enrolled in the study |
|  | Have you gotten any feedback from the school? |
|  | Is there something we should do different in the next study? |

# Children

Conducted subsequent to baseline testing and introduction to the vest.

| Objective | Theme question | Supplementary questions |
| --- | --- | --- |
| F: To investigate the feasibility and practicality of the test situation. Specifically, the children’s ability to switch between different settings (observation in class, testing and fitting of the vest) | Was it okay that I was in your class? |  |
|  | Do you know why we are together here? | And what we are going to do today? |
|  | What do you think about the vest?  Was it difficult to choose between the ones with small or big balls? |  |
| G: To investigate the relevance and usability of the selected outcome measures. | How did you feel about doing the task´s (tests) that you had to do? | - What did you think about them?  - Was it too difficult or was it okay? |
|  | Pulse-measuring devices:  Which one did you like best? |  |

Conducted subsequent to follow up testing:

| Objective | Theme question | Supplementary questions |
| --- | --- | --- |
| E: To investigate experiences of the children using the vest. | What did you think of the vest? | - Did you use it every day?  - at home/ in school?  - What did you friends say to you using the vest? |
|  | What did you think about using the vest? | - Did it help you? |
|  | Is there anything that could make the vest better? | Should we do something different? |

# Parents

Conducted by phone, twice. First subsequent to the call informing them about their child’s in- or exclusion in the study.

| Objective | Theme question | Supplementary questions |
| --- | --- | --- |
| B: To investigate the relevance to parents of the text in the introduction materials, recruitment material and questionnaire. | Were the materials understandable? | - Did you find it relevant that … (the psychologist) gave it for your child?  - Do you believe that you know what it entails to participate? |
|  | Were the questions in the questionnaire relevant and understandable? | - Was it easy to fill out?  - Is there anything else you think we should ask about? |
|  | Do you have any additional questions regarding the project? | - Regarding participations  - Regarding expectations |
| C: To investigate the information process with parents, regarding participation in the study. If included; inclusion in control or intervention group. | What do you think about me calling and like this, to follow up on the questionnaire? |  |
|  | Do you know why your child is included/not included in the study? |  |
|  | If control group: Do you understand why your child will have to wait before trying the vest? |  |

The second phone interview was performed the day before follow-up.

| Objective | Theme question | Supplementary questions |
| --- | --- | --- |
| D: To investigate the usability of parental diaries. | How did it work to fill out the dairy? | - Did you use the dairy every day?  - If not: why? |
|  | Did the questions support a conversation with your child regarding the use of the vest? | - How?  - What did you talk about |
|  | How was it to register if the child received any treatment? | Was it clear what should be written regarding treatments? |
| E: To investigate experiences of the children using the vest. | Did the study live up to your expectations? | - Is there something we should do different from this point forward? |

# Teachers

Conducted subsequent to follow up testing of the child

| Objective | Theme question | Supplementary questions |
| --- | --- | --- |
| A: To investigate the recruitment process and distribution of recruitment material | Did the study live up to your expectations? | - Based on the materials and information given by the psychologist. |
| E: To investigate experiences of the children using the vest. | What do you think about his/hers use of the vest? | - Did he/she use it every day?  - Did it have any effect on his/her participation in school? |
| F: To investigate the feasibility and practicality of the test situation. | Was it okay that I did the observation in class? | - Did it disturb the class?  - You or the children? |
